# Oncogenic switching of hypoxia signalling pathways

**DOI:** 10.1186/2049-3002-2-S1-O8

**Published:** 2014-05-28

**Authors:** Peter J Ratcliffe

**Affiliations:** 1 Henry Wellcome Building for Molecular Physiology, University of Oxford, OX3 7BN, UK

## 

Tumor hypoxia is strongly associated with an adverse prognosis in cancer, irrespective of treatment modality. Analysis of this association has revealed that hypoxia signalling pathways mediated by hypoxia inducible factor (HIF) are up-regulated in cancer, not only by micro-environment hypoxia, but also by diverse oncogenic signal pathways. Pan-genomic analysis of the HIF transcriptional cascade has demonstrated the massive extent of its actions on gene expression at multiple levels. Direct HIF targets include not only coding RNAs, but regulatory long non-coding RNAs, micro-RNAs and translational control proteins. Physiological pathways that are targeted by the HIF system are not restricted to those with direct actions in oxygen homeostasis, but include biosynthetic metabolic pathways as well as molecules acting on cell differentiation, proliferation, migration and survival decisions. Interestingly, isoform/isoenzyme specific patterns of metabolic gene expression that are induced by HIF map well onto those defined in rapidly proliferating normoxic tumor cells, which are also directly targeted by oncogenic pathways operating in parallel. The implication of oncogenic switching of these massive ‘hard-wired’ parallel physiological pathways for cancer phenotypes will be discussed.

